# Supplementary material for: Evaluation of a diagnostic device, CL Detect rapid test for the diagnosis of new world cutaneous leishmaniasis in Peru
Source: PLoS Negl Trop Dis. 2023 Mar 13;17(3):e0011054. doi: 10.1371/journal.pntd.0011054 (PMC10010545; doi:10.1371/journal.pntd.0011054)
Supplement: S1 File — (DOCX) [file pntd.0011054.s001.docx]

# **S1 File. Supplemental Methods**

**Performance of CL Detect Rapid Test.**

Freely circulating antigens in the sample will react with a colloidal gold-conjugate of a monoclonal antibody to TSA. These 2 entities will form an antigen-antibody complex that will flow vertically up the stick until it encounters an immobilized detection zone (the TSA test line) containing an unconjugated rabbit polyclonal antibody to TSA. At the TSA test line, an accumulation of color from the colloidal gold will indicate a positive sample, denoting the presence of *Leishmania* parasites in the lesion sample. No accumulation of color at the TSA test line equates to a negative reading. Satisfactory performance of the assay was confirmed by the appearance of color at the control line.

**Unanticipated Adverse Device Effects.**

As defined in 21 CFR 812.3, a UADE is any serious adverse effect on health or safety or any life-threatening problem or death caused by, or associated with, a device, if that effect, problem, or death was not previously identified in nature, severity, or degree of incidence in the investigational plan or application (including supplementary application), or any other unanticipated serious problem associated with a device that relates to the rights, safety, or welfare of participants. UADEs were also to be reported.

**Prior and Concomitant Therapy.**

Current treatments (ongoing on the date of consent) for leishmaniasis and current medication use (name of medication, dose, and start date) were reported. Subjects who received treatment for leishmaniasis within the last 2 months prior to signing consent were to be excluded from the study.

**Primary Diagnostic Analysis.**

The performance of CL Detect Rapid Test (sensitivity and specificity of CL Detect Rapid Test) was determined by comparing with the gold standard, microscopy of stained lesion samples for identification of *Leishmania* amastigotes. This was done using dental broach and lancet scraping samples separately for all analyses. The following definitions applied to the calculation of assay sensitivity and specificity: **True positive**: Positive by both the CL Detect Rapid Test and the reference method; **False positive**: Positive by the CL Detect Rapid Test but negative for the reference method; **True negative**: Negative by both the CL Detect Rapid Test and the reference method and **False negative**: Negative by the CL Detect Rapid Test but positive for the reference method. **Sensitivity** was calculated as the number of true positives divided by the sum of the number of true positives plus the number of false negatives multiplied by 100%, along with 2-sided exact binomial 95% CI (Clopper-Pearson). Also, sensitivity and specificity were determined by individual *Leishmania* species. Determination of Sample Size **Specificity** was calculated as the number of true negatives divided by the sum of the number of true negatives plus the number of false positives multiplied by 100%, along with 2-sided exact binomial 95% CI. In addition, the **false positive rate** (α, type 1 error, calculated as 1-specificty multiplied by 100%) and **false negative rate** (β, type 2 error, calculated as 1-sensitivity) was also presented, along with 2-sided exact binomial 95% CIs. Secondary Analyses including Adverse Events and Unanticipated Adverse Device Effects, All AEs and UADEs were to be tabulated and summarized.

**Safety parameters adverse events (AEs)**

Adverse events were recorded since CL lesions were sampled using the dental broach and by scraping with a sterile lancet. The use of any of these methods may produce local reactions, including pain, bleeding, erythema, swelling, or secondary infections at the sampling site. Ecchymosis or hematomas may also occur. The following conditions or events were expected to occur in participants enrolled in this study, not necessarily as a result of the sampling or use of the CL *Detect* Rapid Test, but simply as a result of CL itself: erythema, local edema, lymphadenopathy, and secondary infection. Prior to the participant’s departure from the clinic, the study team verified whether the participant has experienced any AEs. All participants were instructed to either return to the study site immediately, or otherwise contact investigators, should they experience signs/symptoms of AEs.

**Ethics approval and consent to participate.**

The study was reviewed and approved by the NAMRU-6 IRB (date: 09 May 2016); Universidad Peruana Cayetano Heredia (date: 14 July 2016), Regional Health Directorate (DIRESA Madre de Dios) (date: 18 January 2018); Hospital Santa Rosa de Puerto Maldonado, Madre de Dios (Date 28 November 2018) and the Ministry of Health of Peru Regulatory Office (OGITT-INS) (date: 29 August 2018, Letter # 1172-2018). Written informed consent was obtained from all study participants. This trial was conducted in accordance with the ethical principles of Good Clinical Practices (GCP), according to the International Conference on Harmonization (ICH) Harmonized Tripartite Guideline.
