# Supplementary figures and images for: Evaluation of a diagnostic device, CL Detect rapid test for the diagnosis of new world cutaneous leishmaniasis in Peru
Source: PLoS Negl Trop Dis. 2023 Mar 13;17(3):e0011054. doi: 10.1371/journal.pntd.0011054 (PMC10010545; doi:10.1371/journal.pntd.0011054)

S STARD diagram to report flow of participants through the study


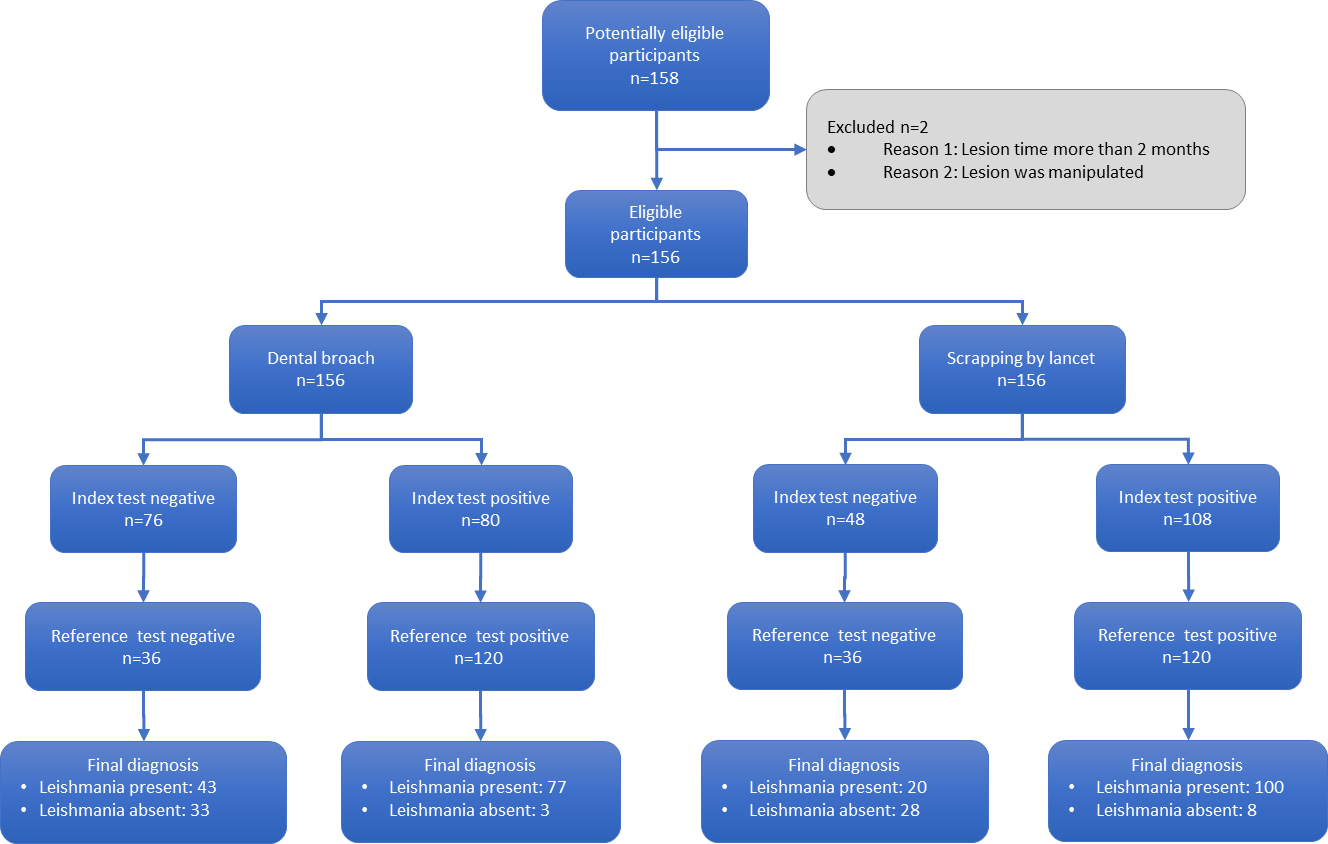

Supplement: S3 File — (DOCX) [file pntd.0011054.s003.docx]
