# Supplementary material for: Evaluation of a diagnostic device, CL Detect rapid test for the diagnosis of new world cutaneous leishmaniasis in Peru
Source: PLoS Negl Trop Dis. 2023 Mar 13;17(3):e0011054. doi: 10.1371/journal.pntd.0011054 (PMC10010545; doi:10.1371/journal.pntd.0011054)
Supplement: S1 Table — (DOCX) [file pntd.0011054.s004.docx]

**S1 Table:** Microscopy by PCR for CL Detect Rapid Test (Collected by Dental Broach and Collected by Scraping) Positive and negative Subjects

| **PCR** | **Microscopy** | |
| --- | --- | --- |
|  | **Positive** | **Negative** |
| **Positive** | 77 | 0 |
| **Negative** | 0 | 3 |

**Microscopy by PCR for *CL Detect* Rapid Test (Collected by Dental Broach) Positive Subjects**

**Microscopy by PCR for *CL Detect* Rapid Test (Collected by Dental Broach) Negative Subjects**

| **PCR** | **Microscopy** | |
| --- | --- | --- |
|  | **Positive** | **Negative** |
| **Positive** | 43 | 0 |
| **Negative** | 0 | 3 |

**Microscopy by PCR for *CL Detect* Rapid Test (Collected by Scraping) Positive Subjects**

| **PCR** | **Microscopy** | |
| --- | --- | --- |
|  | **Positive** | **Negative** |
| **Positive** | 100 | 8 |
| **Negative** | 0 | 0 |

**Microscopy by PCR for *CL Detect* Rapid Test (Collected by Scraping) Negative Subjects**

| **PCR** | **Microscopy** | |
| --- | --- | --- |
|  | **Positive** | **Negative** |
| **Positive** | 20 | 25 |
| **Negative** | 0 | 3 |
