# Supplementary material for: Evaluation of a diagnostic device, CL Detect rapid test for the diagnosis of new world cutaneous leishmaniasis in Peru
Source: PLoS Negl Trop Dis. 2023 Mar 13;17(3):e0011054. doi: 10.1371/journal.pntd.0011054 (PMC10010545; doi:10.1371/journal.pntd.0011054)
Supplement: S2 Table — (DOCX) [file pntd.0011054.s005.docx]

# **S2 Table. Comparison of Results of All Studies by Different Assay Conditions**

| **Protocol Number** | **S-12-14** | **S-12-19** | **LRDD- PERU-1** | **LRDD-PERU-2** |  |  |
| --- | --- | --- | --- | --- | --- | --- |
|  | **Tunisia** | **US** | **Peru All species** | **Peru All species** | **Peru All species** | **Peru** |
|  | ***L. (L.) major*** | **Non-endemic** |  |  |  | ***L. (V.) braziliensis*** |
|  | **N=168** | **N=150** | **N=26** | **N=156** | **N=156** | **N=106** |
| Sample collection | Dental Broach | Dental Broach | Dental Broach | Dental Broach | Lancet Scraping | Lancet Scraping |
| Assay condition | per label | per label | per label | modified^a^ | modified | modified |
| True Positive, n (%) | 149 (88.7) | 0 (0.0) | 6 (23) | 77 (49.4) | 100 (64.1) | 77 (72.6) |
| True Negative, n (%) | 16 (9.5) | 144 (96.0) | 6 (23) | 33 (21.2) | 28 (17.9) | 11 (10.4) |
| False Positive, n (%) | 3 (1.8) | 6 (4.0) | 0 (0) | 3 (1.9) | 8 (5.1) | 6 (5.7) |
| False Negative, n (%) | 0 (0.0) | 0 (0.0) | 14 (54) | 43 (27.6) | 20 (12.8) | 12 (11.3) |
| Sensitivity, % | 100 | Not done | 30 | 64.2 | 83.3 | 86.5 |
| Specificity, % | 84.2 | 96 | 100 | 91.7 | 77.8 | 64.7 |
| False positive rate, % | 16 | 4 | 0 | 8.3 | 22.2 | 35.3 |
| False positive rate, % | 5.3 |  | - | - | - | - |
| False negative rate, % | 0 | 0 | 60 | 35.8 | 16.7 | 13.5 |
| ^a^The modified method included increasing the extraction time to 20 minutes and applying twice the amount of extract to the test strip (40 μL). | | | | | | |
