# Supplementary material for: Evaluation of a diagnostic device, CL Detect rapid test for the diagnosis of new world cutaneous leishmaniasis in Peru
Source: PLoS Negl Trop Dis. 2023 Mar 13;17(3):e0011054. doi: 10.1371/journal.pntd.0011054 (PMC10010545; doi:10.1371/journal.pntd.0011054)
Supplement: Attachment — Submitted filename: CL Detect_response_MD_oct2022.pdf [file pntd.0011054.s009.pdf]

October 21st, 2022
Editorial Board Member
Scientific Reports

Dear Editor,

We appreciate your support and contributions as well as those of the reviewers to improve the quality of our manuscript entitled "Evaluation of a diagnostic device, CL Detect Rapid Test for the diagnosis of new world cutaneous leishmaniasis in Peru".

Kindly find below an updated point-by-point response to the reviewers' comments. We hope that our responses will be satisfactory and the manuscript will be accepted for publication. Please feel free to reach me if there are any additional questions.

Sincerely,

Christie Joya, MD
Lieutenant Commander, Medical Corps,
US Navy Infectious Disease Physician
Naval Medical Center Portsmouth
620 John Paul Jones Circle
Portsmouth, Virginia 23708

Reviewer comments

- Reviewer #3: -   The authors should define the Leishmania species that have initially worked with when stating the objective.
  - The blade used to collect samples by the lancet should be specified.
    Response: We have added this information on line 148:

  - Describe the exact procedure for taking the tissue scraping.
    Response: We have added the information Lancet scraping was performed as follow, the lesion of the patient was cleaned using an alcohol gel pad, then the crust was removed with the blunt end of the lancet and the tissue was obtained from internal border and center of the lesion. Page 7, lines 155-157.

- Reviewer #3: -   Justification for performing such time-consuming work should be clarified. This technique is not a rapid and easy approach to the selection of proper and timely therapy. Conversely, it is a tedious, labor-intensive, cumbersome, and costly procedure. In reality, any conventional diagnostic method should be performed quickly with acceptable sensitivity. We should not neglect simple conservative methods which are achieved through multiple global efforts to be replaced by frustrating, complicated, over-priced, and non-applicable approaches. These techniques are not pertinent and recommended in remote areas of tropical regions with low-income countries where this complex disease is present.
  Response: We appreciate the insight; however, we do not fully agree with the entirety of the reviewer's statement. Conventional diagnostic methods (microscopy) for leishmania are cumbersome in field CL settings in Peru and other regions in South America. Staff in these regions are not fully trained, do not have time availability for accurate microscopy assessment and sometimes they do not even have access to microscopes. Therefore, there is still a need for simpler approaches that can be implemented in field settings. It is true that the cost is a major limitation; however, without studies that evaluate the diagnostic performance of these tests, it is very unlikely that further development of such methods will be done.
  It is also important to highlight that the CL-Detect modifications using a lancet scrapping sample is less invasive and painful for the patients compared to the cytology brush. Furthermore, it avoids the need of local anesthesia.
  Finally, although a previous paper (PMC8797207) does not recommend its use in Ethiopia, the simple modifications introduced in our study to the CL-detect have improved the performance of this method in field conditions (health sites in Puerto Maldonado, Madre de Dios).

- The authors should present the background information about the *Leishmania* spp. that causes CL in the area. As the authors well pointed out, more than 20 *Leishmania* species are causative. Most of them cause CL.

- L 67, 350 million people at risk should be over1 billion…(WHO-Leishmaniasis home).
  Response: Corrected as suggested

- L64, for cutaneous leishmaniasis, presents the abbreviation form (CL) and excludes the CL abbreviation in L72.
  Response: Corrected as suggested

- In vitro should be italicized (L69 and other places).
  Response: Corrected as suggested

- L94 and many places Leishmania species should be italicized.

  Response: Corrected as suggested

- In the paragraph L16-121, there are several techniques used to detect the Leishmania organism, but depending on the species, the lesion sampling stage, personnel, and supplies the sensitivity of these tests are diverse.

  Response: Thank you for the comment
