# Supplementary material for: Evaluation of a diagnostic device, CL Detect rapid test for the diagnosis of new world cutaneous leishmaniasis in Peru
Source: PLoS Negl Trop Dis. 2023 Mar 13;17(3):e0011054. doi: 10.1371/journal.pntd.0011054 (PMC10010545; doi:10.1371/journal.pntd.0011054)
Supplement: Attachment — Submitted filename: PNTD_D_22_00601.docx [file pntd.0011054.s007.docx]

Grogl et al., evaluates a diagnostic tool for CL commonly in Peru, which is commonly used in the Old World. This tool can help supplement current diagnostic tools and can be easily applied in the field. The authors should compare the sensitivity/specificity against qPCR and not only against microscopy through the manuscript.

Line 24. Briefly explain clearly why the need of a modified test in the abstract. This is not very clear/mentioned in the abstract.

Line 24-26. Add the different collections methods for the samples (scrapping and dental broach) as this is also a main difference between the modified and original test.

Line 30: did you compared to the non-modified CL Detect Rapid test with the current set of participants? If not, why not? It would be worth to add a paragraph explaining why not the comparison in your cohort in the discussion.

Line37: qPCR is very sensitive technique for identification of leishmania parasites (kDNA). Perhaps rephase this paragraph to show percentages against both qPCR and microscopy compared to your modified test and not only compared to microscopy.

Line 40: Your results showed a high percentage of false positive rate for the modified assay, then to make a further decision for patient treatment an additional diagnostic test would be needed. Please rephrase the paragraph in line 41.

Line 51: add sensitivity range % after low sensitivity of ….. “however, in the New World the FDA-cleared device has low sensitivity….”

Figure 1: I would strongly suggest the authors to test the sensitivity with other Leishmania *spp*. (ex. L. (V.) braziliensis) found in Peru and not only against *Leishmania major* from the Old-world.

Line 112: “to evaluate its viability” this it’s a bit confusing as before you are only talking about sensitivity of the assay using different scrapping/collection methods and did not mention anything about viability before in the manuscript… please correct/rephrase this sentence.

Line 170: Add more details regarding the qPCR method used in addition to the reference (14). This would help the reader.

Line 286-288: Regarding the following paragraph: *“There were 8 false positives by the modified CL Detect compared with microscopy when samples were collected by lancet scraping; however, these were all positive by the more sensitive kDNA PCR”.* This for me means that you test is more sensitive/specific as it is comparable to a more sensitive diagnostic technique which is qPCR. I understand that in the field the golden standard for CL diagnostic is microscopy but the comparison here and through the manuscript for both sensitivity and specificity should be done against both microscopy and qPCR.

Line 315: discuss briefly why the lower sensitivity for of the test in the New World compared to Old World.
