# Supplementary material for: Evaluation of a diagnostic device, CL Detect rapid test for the diagnosis of new world cutaneous leishmaniasis in Peru
Source: PLoS Negl Trop Dis. 2023 Mar 13;17(3):e0011054. doi: 10.1371/journal.pntd.0011054 (PMC10010545; doi:10.1371/journal.pntd.0011054)
Supplement: Attachment — Submitted filename: CL Detect Manuscript Final Draft- Reviewer comments .docx [file pntd.0011054.s006.docx]

**~~Title:~~ Evaluation of a diagnostic device, CL *Detect*™ Rapid Test for the diagnosis of new world cutaneous leishmaniasis**

Max Grogl^4¶^, Christie A. Joya^1*¶^, Maria Saenz^2&^, Ana Quispe^3&^, Luis Angel Rosales^2&^, Rocio del Pilar Santos^2&^, Maxy B. De los Santos^1&^, Ngami Donovan^5&^, Janet H. Ransom^5&^, Ana Ramos^3&^, Elmer Llanos Cuentas^3¶^

^1^Department of Parasitology, Naval Medical Research Unit No. 6, Callao, Lima, Peru

^2^Vysnova, Lima, Peru

^3^Universidad Cayetano Heredia, Lima , Peru

^4^ Naval Medical Research Center, Silver Spring, MD, USA

^5^ Fast-Track Drugs & Biologics, LLC, Poolesville, MD, USA

*[christie.a.joya.mil@mail.mil](mailto:christie.a.joya.mil@mail.mil) (CJ)

^¶^These authors contributed equally to this work.

^&^These authors also contributed equally to this work.

# **Abstract**

**Background**

Cutaneous leishmaniasis (CL) is a neglected disease and a public health problem in Latin America. The primary objective of this study was to determine the sensitivity and specificity of the FDA-cleared CL *Detect*™ Rapid Test in Peru, using modified test procedures rather than the instructions-for-use, by 1) increasing the extraction time and 2) increasing the volume of the sample added to the test strip. CL *Detect*™ Rapid Test results were compared against the gold standard, microscopy, for the diagnosis of CL in ulcerated lesions.

**Methodology**

Participants were patients who presented for medical consultation due to a suspected CL lesion. Four samples from the index lesion were collected using a dental broach, per package insert, and lancet scraping and tested by the modified CL *Detect*™ Rapid Test, microscopy, and PCR.

**Principal findings**

A total of 156 subjects were eligible and evaluated. The modified CL *Detect*™ sensitivity was higher in specimens obtained by scraping (83.3%) than those from dental broach (64.2%). The specificity was lower in scrapings (77.8%) with a false positive rate of 22.2% compared with dental broach samples (91.7%) with a false positive rate of 8.3%. However, molecular analysis showed that all 8 false positive scrapings (those positive by modified CL Detect and negative by microscopy) were positive by kDNA-PCR.

**Conclusions**

These modifications to the package insert that resulted in a diagnostic sensitivity (83.3%) comparable to microscopy for species found in Peru may enable earlier anti-leishmanial drug treatment decisions based on a positive result from the CL *Detect*™ Rapid Test alone until further diagnostic tests can be performed.

# **Author Summary**

Cutaneous leishmaniasis is commonly referred to as the most neglected of the neglected tropical diseases. While it is rarely deadly, it can cause chronic non-healing ulcers and scarring that can lead to contractures and social stigma. Due to the toxicity of the treatments for this disease, most ministries of health require a diagnosis prior to its initiation. Unfortunately, diagnosis can be quite difficult, as the gold standard of microscopy requires highly trained personnel. The rapid, point-of-care diagnostic device CL *Detect*™ has been a viable option for Old World cutaneous leishmaniasis; however, in the New World the FDA-cleared device has low sensitivity. In this study, three simple changes (sample collection by the standard method of scraping using a lancet, increased extraction time, and increased sample volume) to the instructions were implemented and showed a similar diagnostic sensitivity to microscopy for the diagnosis of cutaneous leishmaniasis in Peru.

# **Introduction**

Cutaneous leishmaniasis is a parasitic disease that causes disfiguring lesions. It is mainly found in the tropical and subtropical areas in the Middle East, southwest Asia, the Mediterranean coast, sub-Saharan Africa, Mexico, and Central and South America, and it is spread through the bite of infected sand flies. It places 350 million people at risk worldwide, with 1.5 million new cases emerging annually, including U.S. service members serving abroad (1, 2).

The CL *Detect*™ Rapid Test is a qualitative, in vitro immunochromatographic assay for the rapid detection of *Leishmania* species antigen in ulcerative skin lesions. The test is intended for use with dental broach samples from ulcerative skin lesions that are obtained from patients with suspected cutaneous leishmaniasis (CL). The test targets the peroxidoxin antigen of *Leishmania* species that may cause CL and is intended to aid in the diagnosis of CL as it must be interpreted within the context of relevant epidemiologic, clinical and laboratory findings (3).

The *CL Detect*™ was developed by the U.S. Army in partnership with InBios International Inc. Seattle, WA, United States of America, the Institut Pasteur de Tunis in Tunisia, Walter Reed Army Institute of Research, and the U.S. Army Small Business Innovation Research Program (SBIR). This program allowed InBios, a small, U.S. medical device manufacturer, the opportunity to provide an innovative research and development solution in response to a critical Army need; the rapid diagnosis of cutaneous leishmaniasis in an operational setting. It received clearance from the Food and Drug administration (FDA) in 2014.

*CL Detect*™ works when freely circulating antigens in the sample react with a colloidal gold-conjugate of a monoclonal antibody to Thiol-specific antioxidant (TSA) (4). These two entities will form an antigen-antibody complex that will flow vertically up the test stick until encountering an immobilized detection zone (the TSA test line) containing an unconjugated rabbit polyclonal antibody to TSA. At the TSA test line, an accumulation of color from the colloidal gold will indicate a positive sample, denoting the presence of *Leishmania* parasites in the lesion sample.

The approval of the *CL Detect*™ Rapid Test was based on clinical trials in Tunisia (Old World CL species) and for specificity in the United States (ulcerated lesions caused by other etiologies) (3, 5). However, the sensitivity of diagnostic tests is known to depend on the species of *Leishmania* and on the amount of amastigotes (a thiol-specific antioxidant source, TSA antigen) found in the CL lesion (4, 6). The parasite load of lesions from Old World species (*Leishmania major*) tend to be higher than those found in the New World (example *L. peruviana* and *L.* *braziliensis* infections) (7-10). Furthermore, Fig 1 shows that the color intensity on the TSA test line of the CL *Detect*™ Rapid Test strip correlates with the number of promastigotes, as the TSA antigen is present in both amastigotes and promastigotes (3). Thus, it is reasonable to believe that the sensitivity of the FDA-cleared CL *Detect*™ Rapid Test can be lower in New World *Leishmania* endemic regions (like Peru) where the number of parasites in the tissue is lower compared to Tunisia (11).

**Fig 1.** **CL *Detect*™ Test Strip Intensity Compared to Number of Promastigotes.** The intensity of the TSA Test Line increases with increasing parasite number. Lanes 1-7 show *Leishmania major* promastigotes from 0, 10, 100, 500, 10^3^, 10^4^, 10^5^ parasites per assay (20 µl sample).

To expand the diagnostic options for CL, the CL *Detect*™ Rapid Test (CL Detect) and the Loopamp™ *Leishmania* Detection Kit (Loopamp) were evaluated in Surinam where CL is a serious health problem (12). Not surprisingly, the study showed that the CL Detect had a very low sensitivity compared to microscopy (36.7%) or PCR (35.8%), due to a high number of false negative results. Thus, the authors concluded CL Detect was not likely to be a good replacement for the routine diagnostic procedure for CL in Suriname.

Likewise, in a 2014-2016 study conducted by NAMRU-6 and the Instituto de Medicina Tropical Alexander von Humboldt (IMTAvH), in Peru (PERU-01, unpublished) we found the sensitivity of the CL *Detect*™ Rapid Test was only 50% using the device instructions.

In the present study (PERU-02), we hypothesize that implementing two simple modifications of the test procedure described in the device instructions the sensitivity of the FDA-cleared CL *Detect*™ Rapid Test could drastically increase and it will suffice to deliver a diagnosis in a short time, accelerating patient’s access to treatment in remote endemic areas and real field situations. Additionally, we compared sample taking with the dental broach (per package insert) with the traditional lancet scraping to evaluate its viability.

# **Methods**

**Study population**

Adults (≥18 years of age) presenting to Universidad Peruana Cayetano Heredia (UPCH) and the regional health posts and centers in the Madre de Dios region of Peru (Jorge Chávez, Milenio, Iberia and Santa Rosa) for evaluation of skin lesions consistent with CL were invited to participate in the study. Inclusion criteria included an index lesion that was primarily ulcerative in appearance (not purely verrucous or nodular), present for less than 2 months, without clear evidence of cellulitis and located in a suitable area for collecting samples by both dental broach and lancet scraping, as well as being capable of understanding and complying with the protocol. Subjects were excluded if they had received anti-leishmanial treatment in the 2 months prior to presentation or if the lesion had evidence of manipulation. Enrollment took place between October 2018 and September 2019, terminating when predefined sample size was met.

**Sample Collection**

After obtaining informed consent and screening participants for eligibility, an index lesion was identified and sampled on the internal lesion border in the following order: 1) dental broach sample for modified CL *Detect*™ Rapid testing; 2) lancet sample for microscopy to assess the presence of *Leishmania* amastigotes; 3) lancet sample for PCR; and 4) when possible (depending on the size of the lesion), a sample from a different site of the same lesion, obtained by scraping by lancet for use with the modified CL *Detect*™ Rapid Test. Adverse events (AEs) and unanticipated adverse device events (UADEs) were collected in the clinic starting with the time of the collection of the first lesion sample with the dental broach and until the subject left the clinic after study procedures were completed.

Participant demographics including age, gender, and ethnicity were collected. The length of time between initial presence of index lesion and time of lesion sampling, history of leishmaniasis including any prior (past 2 months) or current treatments for leishmaniasis, current medication use (name of medication, dose, and start date), and the anatomical location of the lesion were also captured.

**Modified CL *Detect*™ Rapid Test**

The following modifications to the device instructions were used: 1) increase extraction time in lysis buffer from 5 to 10 minutes to 20 to 30 minutes; and 2) increase from 20 μL to 40 μL in the volume of the sample to add to the test strip in the area beneath the arrow. Lesion tissue obtained via dental broach sampling was placed in the lysis buffer for 20 minutes but not more than 30 minutes, then 40 μL directly applied onto the reservoir pad of the CL *Detect*™ Rapid Test dipstick. The scraping taken from a different site of the same lesion was also tested with the modified CL *Detect*™ Rapid Test.

Satisfactory performance of the assay was confirmed by the appearance of color at the control line. The result of the test was recorded and photographed. The intensity of the “signal” (color at the TSA test line) was compared with a color intensity chart (scale 0 to 15) provided by InBios and the results were recorded.

**Microscopy**

Microscopy slides were prepared at the enrolling sites, smears were made by spreading the scraping material to make a thin preparation on two separate slides that were air dried, fixed with methanol and stained with Giemsa solution or Diff-Quick stain. The presence of parasites in the index lesion was determined by performing parasite counts on scraping samples by clinical staff at UPCH. Amastigotes in macrophages were counted according to the World Health Organization (WHO) guidance for grading parasite density using a 10x eyepiece and 100x oil objective lens (13). The results were not shared with the enrolling sites laboratory personnel conducting the CL *Detect*™ test. A third slide was prepared and retained at the NAMRU-6 Puerto Maldonado site for diagnosis of the patients who participated in the study there. Only amastigotes with clearly defined and correctly stained nuclei, kinetoplasts, and cytoplasm and cell membranes were counted. The average parasite density was then determined according to the WHO reference standard and the results were recorded.

***Leishmania* diagnostic methodology by kDNA-PCR**

DNA extraction was carried out using the DNeasy Blood & Tissue Kit (Qiagen) following the manufacturer recommendations. DNA detection by kDNA-PCR was performed using 4 µL DNA and following the method targeting a region of *Leishmania* minicircle for amplification of CL-causing species as described elsewhere (14). This PCR was used as a highly sensitive molecular test to compare to the performance of CL *Detect*™ Rapid Test and microscopy. The PCR assay was performed by NAMRU-6 Lima laboratory technicians. These personnel did not have access to the results generated by either the enrolling sites laboratory personnel nor the microscopists trained for the study.

**Determination of *Leishmania* species by FRET-based Nested Real-Time PCR**

For species identification, a FRET-based Nested Real-Time PCR targeting the mannose phosphate isomerase (MPI) and 6-phosphogluconate dehydrogenase (6PGD) genes was performed and melting peaks patterns were compared with those from reference strains *L. (V.) braziliensis* (MHOM/BR/84/LTB300), *L. (V.) peruviana* (MHOM/PE/87/PAB2880), *L. (V.) guyanensis* (MHOM/BR/75/M4147), *L. (V.) panamensis* (MHOM/PA/71/LS94), *L. (V.) lainsoni* (MHOM/PE/88/BAB1730), *L. (V.) amazonensis* (MHOM/BR/73/M2269) and *L. (V.) mexicana* (MHOM/BZ/82/BEL21) following conditions previously established (15).

**Statistical methods**

Summary statistics including number and percentage for discrete variables, and number, mean, standard deviation, median, minimum, and maximum values for continuous variables, and by-participant displays of all data collected were estimated. The evaluable analysis set included all subjects for whom the modified CL *Detect™* Rapid Test and microscopy was performed and a result was reported as either positive or negative. The safety analysis set included all subjects who underwent any baseline procedures on Study Day 1 including preparation of the lesion site and sample collection from the index lesion. The performance of modified CL *Detect*™ Rapid Test (sensitivity and specificity) was compared with the gold standard, microscopy of a stained lesion sample for identification of *Leishmania* amastigotes. Sensitivity was calculated as the number of true positives divided by the sum of the number of true positives plus the number of false negatives. Specificity was calculated as the number of true negatives divided by the sum of the number of true negatives plus the number of false positives.

**Primary diagnostic analysis**

The performance of modified CL *Detect*™ Rapid Test (sensitivity and specificity) along with 2-sided exact binomial 95% CI was determined by comparing with the gold standard, microscopy of stained lesion samples for identification of *Leishmania* amastigotes. This was done using dental broach and scraping by lancet samples separately for all analyses.

**Determination of sample size**

The sample size determination of sensitivity at this site in Peru was based on the prior pilot study in Peru using the modified procedure, it was assumed that the point estimate for test sensitivity will be at least 80% (with a false negative rate = 20%). Using the approach for determining samples sizes for diagnostic tests by Malhotra, the minimum N that will have a lower 95% CI that is least 70% was estimated in 90 subjects (16).

**Ethics statement**

The study protocol (NAMRU6.2015.0009) was reviewed and approved by the Institutional Review Boards (IRBs) of NAMRU-6, and Universidad Peruana Cayetano Heredia and received approval from the Regional Health Directorate of Madre de Dios and from the Ministry of Health of Peru Regulatory Office (OGITT-INS) and registered at Clinicaltrials.gov (NCT03762070). Written informed consent was obtained from all study participants. This trial was conducted in accordance with the ethical principles of Good Clinical Practices (GCP), according to the International Conference on Harmonization (ICH) Harmonized Tripartite Guideline.

# **Results**

**Clinical Features**

Written consent was obtained from a total of 158 subjects. All but two (n=156, 98.7%) of the consented subjects were eligible, underwent all testing procedures, and completed the study; zero ~~(0.0%)~~ participants terminated early.

Of the 156 subjects who participated in the study, 83.3% were male, the median age was 31.5 years (range 18 to 84), and all but one was Hispanic or Latino (Table 1). Most lesions were found on the legs (41.0%) and arms (23.1%) (Table 1). Per kDNA-PCR analysis, only 3 lesions were negative for leishmaniasis, and of the 153 positive, 67.9% were *L. (V.) braziliensis*, 13.5% were *L. (V.) peruviana*, 9.6% could not be identified by FRET-based Nested Real-Time PCR and the remaining were *L. (V.) guyanensis* (4.5%), and *L. (V.) lainsoni* (2.6%). The mean age of lesions from the time that they were first noticed by the study subject to the time of testing was 35.7 days (range 3 to 61 days).

**Table 1. Demographic and Other Baseline Characteristics**

| **Demographics – Evaluable Subjects (N=156)** | |
| --- | --- |
| **Sex** |  |
| Female, N (%) | 26 (16.7) |
| Male, N (%) | 130 (83.3) |
| **Age (years at date of consent)** |  |
| Mean (SD) | 35.3 (14.5) |
| Median | 32 |
| Range | 18 - 84 |
| **Ethnicity-Evaluable subjects** |  |
| Hispanic or Latino, N (%) | 155 (99.4) |
| Not Hispanic or Latino, N (%) | 1 (0.6) |
| **Baseline lesions characteristics-Evaluable subjects (N=156)** | |
| **Primary infecting species (by Nested RT-PCR)** |  |
| *L. (V.) braziliensis* | 106 |
| *L. (V.) guyanensis* | 7 |
| *L. (V.) lainsoni* | 4 |
| *L. (V.) peruviana* | 21 |
| Not identified | 15 |
| Negative for cutaneous leishmaniasis | 3 |
| **Body sites** |  |
| Leg | 64 |
| Arm | 36 |
| Hand | 15 |
| Face | 15 |
| Back | 10 |
| Chest | 7 |
| Foot | 5 |
| Buttock | 2 |
| Head | 1 |
| Abdomen | 1 |
| **Days before sample collection that lesions were first noticed (days)** | |
| Mean (SD) | 35.7 (17.1) |
| Median | 33 |
| Range | 3 - 61 |
| All lesions were ulcerated, none were manipulated, or had cellulitis. | |

**Sensitivity and specificity of modified CL *Detect*™ vs microscopy**

All samples were collected from a single lesion using a dental broach and sterile lancet. Of the 156 specimens, 120 (77%) were positive and 36 (23%) were negative by microscopy. Of the microscopy positive samples, 77 were also positive by modified CL *Detect*™ Rapid Test when the sample was collected with a dental broach but 100 were positive by modified CL *Detect*™ Rapid Test when the sample was collected by scraping using the blunt end of the lancet (Table 2). Of the 36 samples negative by microscopy, 33 were also negative by modified CL *Detect*™ Rapid Test when the sample was collected with a dental broach but fewer, 28, were negative by modified CL *Detect*™ Rapid Test when the sample was collected by scraping-lancet (Table 2).

**Table 2. Modified CL *Detect*^TM^ Rapid Test vs Microscopy Test**

| **Modified *CL Detect*^TM^ Rapid Test** | **Microscopy test** | | | | | |
| --- | --- | --- | --- | --- | --- | --- |
|  | **Dental broach specimens** | | | **Scraping by lancet specimens** | | |
|  | **Positive** | **Negative** | **Total** | **Positive** | **Negative** | **Total** |
| **Positive** | 77 | 3 | 80 | 100 | 8 | 108 |
| **Negative** | 43 | 33 | 76 | 20 | 28 | 48 |
| **Total** | 120 | 36 | 156 | 120 | 36 | 156 |

The modified CL *Detect*™ Rapid Test sensitivity when compared with microscopy was 64.2% when the sample was collected using the dental broach but was higher at 83.3% when collected by lancet (Table 3). However, the false positive rate was higher with lancet specimens at 22.2% compared with 8.3% with dental broach specimens, making the specificity at 77.8% for scraping specimens and 91.7% for dental broach specimens (Table 3).

**Table 3. Modified CL *Detect™* Rapid Test Sensitivity and Specificity Analysis**

| **Modified CL Detect^TM^ Rapid Test** | **N=156** | |
| --- | --- | --- |
|  | **Dental Broach Specimens** | **Scraping by Lancet Specimens** |
| True Positives n (%) | 77 (49.4%) | 100 (64.1%) |
| False Negatives n (%) | 43 (27.6%) | 20 (12.8%) |
| True Negatives n (%) | 33 (21.2%) | 28 (17.9%) |
| False Positives n (%) | 3 (1.9%) | 8 (5.1%) |
| Sensitivity (95% CI) | 64.2% (54.9% - 72.7%) | 83.3% (75.4% - 89.5%) |
| Specificity (95% CI) | 91.7% (77.5% - 98.2%) | 77.8% (60.8% - 89.9%) |
| False positive rate (95% CI) | 8.3% (1.8% - 22.5%) | 22.2% (10.1% - 39.2%) |
| False negative rate (95% CI) | 35.8% (27.3% - 45.1%) | 16.7% (10.1% - 24.6%) |

**Comparison of microscopy with kDNA-PCR**

The modified *CL Detect™ Rapid Test* results versus the combined results of microscopy and the more sensitive kDNA-PCR method were examined. To compare the results, 2 x 2 tables were prepared for modified CL *Detect*™ Rapid Test positive and negative subjects by type of sample collection method (Table 4). Of the 77 positive dental broach samples that were positive by microscopy, all were also positive by kDNA-PCR (Table 4). Seventy-six of the dental broach samples were negative, of which 43 were positive by microscopy and kDNA-PCR, and 73 were positive by kDNA-PCR. This means that 30 samples that were kDNA-PCR positive were microscopy negative (Table 4). The greater sensitivity when samples were collected by lancet scraping compared with the dental broach is supported by the kDNA-PCR data, where 108 samples were positive by modified CL *Detect*™ Rapid Test and kDNA-PCR, of which 8 of these samples were negative by microscopy (Table 4). These 8 samples were considered false positive when compared with microscopy, but were confirmed positive by kDNA-PCR. The lower sensitivity of the microscopy method compared with kDNA-PCR is evident in 25 samples negative for leishmaniasis by both the microscopy method and the modified *CL Detect™ Rapid Test* with scraping-lancet that were positive by kDNA-PCR (S1 Table).

**Table 4a. Modified CL *Detect*^TM^ Rapid Test Sensitivity and Specificity by Species of Samples Collected with Dental Broach – All Subjects**

| **Species** | **N** | **True Positive** | **False Negative** | **True Negative** | **False Positive** | **Sensitivity** | **Specificity** |
| --- | --- | --- | --- | --- | --- | --- | --- |
|  |  |  |  |  |  |  |  |
|  |  | **n (%)** | **n (%)** | **n (%)** | **n (%)** |  |  |
| *L. (V.) braziliensis* | 106 | 61 (57.5) | 28 (26.4) | 14 (13.2) | 3 (2.8) | 68.5% | 82.4% |
| *L. (V.) peruviana* | 21 | 9 (42.9) | 8 (38.1) | 4 (19.0) | 0 (0.0) | 52.9% | 100.0% |
| *L. (V.) guyanensis* | 7 | 5 (71.4) | 2 (28.6) | 0 (0.0) | 0 (0.0) | 71.4% | NA^a^ |
| *L. (V.) lainsoni* | 4 | 2 (50.0) | 2 (50.0) | 0 (0.0) | 0 (0.0) | 50.0% | NA |
| Not identified^b^ | 15 | 0 (0.0) | 3 (20.0) | 12 (80.0) | 0 (0.0) | 100.0% | 100.0% |
| ^a^ NA – cannot be calculated as denominator is zero.  ^b^ Sample was positive by kDNA-PCR but the species could not be identified. | | | | | | | |

Table 1b. Modified *CL Detect*^TM^ Rapid Test Sensitivity and Specificity by Species of Samples Collected by Lancet– All Subjects

| Species | N | True  Positive  n (%) | False Negative n (%) | True Negative n (%) | False Positive n (%) | Sensitivity | Specificity |
| --- | --- | --- | --- | --- | --- | --- | --- |
| *L. (V.)braziliensis* | 106 | 77 (72.6) | 12 (11.3) | 11 (10.4) | 6 (5.7) | 86.5% | 64.7% |
| *L. (V.)peruviana* | 21 | 13 (61.9) | 4 (19.0) | 3 (14.3) | 1 (4.8) | 76.5% | 75.0% |
| *L. (V.)guyanensis* | 7 | 7 (100.0) | 0 (0.0) | 0 (0.0) | 0 (0.0) | 100.0% | NA |
| *L. (V.) lainsoni* | 4 | 2 (50.0) | 2 (50.0) | 0 (0.0) | 0 (0.0) | 50.0% | NA |
| Not identified | 15 | 1 (6.7) | 2 (13.3) | 11 (73.3) | 1 (6.7) | 33.3% | 91.7% |

^a)^	NA – cannot be calculated as denominator is zero.

^b)^	Sample was positive by PCR but the species could not be identified.

**Sensitivity and specificity Modified CL *Detect*™ vs microscopy by species**

Of the two major species of leishmaniasis in Peru, *L. (V.) braziliensis* and *L. (V.) peruviana* were most frequently represented in the study population with 106 *L. (V.) braziliensis* samples and 21 *L. (V.) peruviana* samples (Table 4). Other species included *L. (V.) guyanensis* (7 samples), *L. (V.) lainsoni* (4 samples), and 15 kDNA-PCR positive samples that were not identifiable. The sensitivity and specificity of the modified *CL Detect™ Rapid Test* (microscopy as the standard comparator) by species for samples collected with the dental broach or by lancet scraping are shown in Table 4. As expected from the overall data, the sensitivity was higher for most of the species (with only 4 *L. (V.) lainsoni* samples they were the same) when samples were collected by lancet scraping compared with the dental broach. The sensitivity was 86.5% for *L. (V.) braziliensis*, 76.5% for *L. (V.) peruviana*, and 100% for *L. (V.) guyanensis* for lancet scraping (Table 4). Likewise, the specificity was lower (Table 4). Interestingly, the numbers of true positives for kDNA-PCR samples whose species was not identified by FRET-based Nested Real-Time PCR were 0 for dental broach samples and 1 for scraping samples.

**Analysis of false positives and false negatives**

There were 8 false positives by the modified CL Detect compared with microscopy when samples were collected by lancet scraping; however, these were all positive by the more sensitive kDNA-PCR. In contrast, there were only 3 false positives when samples were collected with the dental broach. The overall false negative rate with scraping samples was 16.7% and with dental broach samples it was 35.8% (Table 3 and Table 2, respectively). Although there were only 4 *L. (V.) lainsoni* positive samples, 2 were false negative by the CL *Detect*™ Rapid Test (Table 4) by either collection method.

**Amastigote densities and modified CL Detect™ Rapid Test line intensities**

As shown in Fig 1, the intensity of the line in the CL *Detect*™ Rapid Test should correlates with the density of *Leishmania* amastigotes in samples. Bubble charts showing the amastigote density versus the *CL Detect™* Rapid Test line intensities for dental broach samples and lancet scraping samples are shown in Fig 2. There was a positive slope in both charts but linear regression R^2^ was low for both samples, but higher for lancet scraping samples. Amastigote densities were also examined by *Leishmania* species. Mean and median densities were highest for *L. (V.) braziliensis* and *L. (V.) peruviana* (Table 5).

**Fig 2. Modified CL *Detect*™ Rapid Test Intensity versus WHO Amastigote Density of Samples Collected by Scraping.** The figure shows bubble charts for dental broach (A) and lancet scrapping (B). The Y-axis shows the CL-detect signal intensity (scale 0 to 15) whereas the X-axis shows the WHO amastigote density (scale 1-6).

**Table 5. WHO Amastigote Density Scale in Microscopy Positive Specimens**

| **Species** | **N** | **Mean** | **SD** | **Median** | **Min** | **Max** |
| --- | --- | --- | --- | --- | --- | --- |
| *L. (V.) braziliensis* | 89 | 2.7 | 1.3 | 3 | 1 | 6 |
| *L. (V.) peruviana* | 17 | 2.6 | 0.8 | 3 | 1 | 4 |
| *L. (V.) guyanensis* | 7 | 1.9 | 0.9 | 2 | 1 | 3 |
| *L. (V.) lainsoni* | 4 | 2.5 | 2.4 | 1.5 | 1 | 6 |
| Not identified^a^ | 3 | 1 | 0 | 1 | 1 | 1 |
| ^a^ Sample was positive by kDNA-PCR but the species could not be identified | | | | | |  |

# **Discussion**

The CL *Detect*™ Rapid Test has 510(k) premarketing approval and was designed to detect the presence of *Leishmani*a. The basis for the approval was from studies conducted in Tunisia where *L. (L.) major* is prevalent and the United States (as a specificity control site). The results of these studies showed that the test sensitivity was 100% in the Old World (Tunisia study) and the specificity was 96% (US study). The overall false positive rate for the combined studies was 5.3% (3). However, post approval studies have shown lower sensitivities, especially in the New World (4, 12).

The FDA approved kit uses specimens collected with a dental broach. This procedure is painful, invasive and demanding when compared to the more common practice to collect samples by scraping, the standard sample collecting method for CL diagnosis worldwide. Use of a dental broach for sample collection usually requires an injection of a local anesthetic for numbing during the sampling procedure. Local anesthesia not only takes time, but entails all the occasionally side effects of an injectable anesthetic especially in children that cannot easily tolerate the pain. The improvement of the sensitivity of the test by the scraping method compared to the more invasive and complicated use of the dental broach is important and can also be explained by the amount of CL lesion tissue sample collected. An increase in the amount of tissue results in a higher number of amastigotes and the amount target antigen present increasing the test sensitivity of the test. This study confirmed that by changing the assay conditions that the overall sensitivity was improved to 64.2% from 30% (12) found under the label assay conditions in the New World.

A further improvement was observed when the samples were collected by lancet scraping at a sensitivity of 83.3% with the sensitivity for *L. (V.) braziliensis* at 86.5% (S2 Table). However, there was a loss in specificity at 77.8% overall and at 64.7% for *L. (V.) braziliensis* with a false positive rate at 22.2% overall and at 35.3% *for L. (V.) braziliensis*. However, all 8 false negative lancet scraping samples were positive by kDNA-PCR. Thus, samples positive by the modified CL *Detect*™ Rapid Test but negative by microscopy were in fact from subjects infected with *Leishmania*. Therefore, the decision to start treatment of a patient based on a positive result from the modified CL *Detect*™ Rapid Test alone is reasonable until such time as further diagnostic tests can be performed.

Results from the modified CL *Detect*™ are available within 30 minutes, a significant improvement over the current process generally used in endemic countries. Diagnosing CL often requires samples to be shipped to and tested at reference central laboratories far from where the patients reside and taking days if not weeks to receive test results. This is important because in many endemic countries like Peru, CL treatment is free but is not provided by the Ministry of Health without a positive diagnostic test. Rapid diagnosis means treatment can begin immediately, people do not have to leave their workplace multiple times, and travel, sometimes times for days, to visit the treatment center.

For the Department of Defense of the United States, the modified CL *Detect*™ will serve as an important asset in the general diagnosis of skin lesions, distinguishing cutaneous leishmaniasis from other important infectious diseases with a similar presentation. It will allow implementation of appropriate treatments, which can reduce the severity of scarring, reduce lost duty time and improve healthcare and morale for U.S. military personnel in numerous areas of operations. Deployed medics will be able to diagnose CL in 98 countries where CL is endemic, including New World species.

# **Acknowledgements**

We are indebted to the patients and Ministry of Health of the Madre de Dios region of Peru, the nurses, Silvia Revilla and Gloria Fuentes, and scientists, Dr. Danette Bishop and Dr. Edward Smith who contributed to the study as well as to Dr. Joan L. Cmarik, Science Advisor/DHP 6.7 Manager, Office of the Principal Assistant for Acquisition US Army Medical Center and Development Command, for the excellent management of D6.7_16_C2_I_16_J9_1527F and support to the project and NAMRU-6. We are also grateful to InBios International Inc., USA for the donation of the CL Detect™ Rapid Test; the company had no role in study design, data collection and analysis, decision to publish, or preparation of the manuscript.

# **Copyright statement**

Some authors of this manuscript are military service members and employees of the U.S. Government. This work was prepared as part of their official duties. Title 17 U.S.C. §105 provides that “Copyright protection under this Title is not available for any work of the United States Government”. Title 17 U.S.C. §101 defines a U.S. Government work as a work prepared by a military service member or employee of the U.S. Government as part of that person’s official duties.

# **Funding Statement**

This study was supported by funding from the US Department of Defense Health Agency 6.7 Intramural Funding.

# **Disclaimer**

The views expressed in this manuscript reflect the results of research conducted by the author and do not necessarily reflect the official policy or position of the Department of the Navy, Department of Defense, nor the United States Government

# **Human Research/Institutional Review Board (IRB) statement**

# The study protocol NAMRU6.2015.0009 was approved by the Naval Medical Research Unit No. 6 Institutional Review Board in compliance with all applicable federal regulations governing the protection of human subjects.”

# **References**

1.	Leishmaniasis in Active Component, Reserve/Guard, and Other Beneficiaries from 2005-2014. 2015 May 14

2.	Status of endemicity of cutaneous leishmaniasis worldwide, 2015: World Health Organization 2017.

3.	CL Detect (TM) Rapid Test for Cutaneous Leishmaniasis [Package Insert]. InBios. Seattle, Washington2014.

4.	De Silva G, Somaratne V, Senaratne S, Vipuladasa M, Wickremasinghe R, Wickremasinghe R, et al. Efficacy of a new rapid diagnostic test kit to diagnose Sri Lankan cutaneous leishmaniasis caused by Leishmania donovani. PLoS One. 2017;12(11):e0187024.

5.	Ben Salah A, Zaatour A, Gharbi A, Bettaieb J, Ghawar W, Khedher A. Clinical Evaluation of CL Detect(TM) Rapid Test for Cutaneous Leishmaniasis: Performance Characteristics Wehn Compared to Smear Microscopy at Multiple Sites ASTMH New Orleans 2014.

6.	Reimao JQ, Coser EM, Lee MR, Coelho AC. Laboratory Diagnosis of Cutaneous and Visceral Leishmaniasis: Current and Future Methods. Microorganisms. 2020;8(11).

7.	Jara M, Adaui V, Valencia BM, Martinez D, Alba M, Castrillon C, et al. Real-time PCR assay for detection and quantification of Leishmania (Viannia) organisms in skin and mucosal lesions: exploratory study of parasite load and clinical parameters. J Clin Microbiol. 2013;51(6):1826-33.

8.	Suarez M, Valencia BM, Jara M, Alba M, Boggild AK, Dujardin JC, et al. Quantification of Leishmania (Viannia) Kinetoplast DNA in Ulcers of Cutaneous Leishmaniasis Reveals Inter-site and Inter-sampling Variability in Parasite Load. PLoS Negl Trop Dis. 2015;9(7):e0003936.

9.	Amorim CF, Novais FO, Nguyen BT, Misic AM, Carvalho LP, Carvalho EM, et al. Variable gene expression and parasite load predict treatment outcome in cutaneous leishmaniasis. Sci Transl Med. 2019;11(519).

10.	Pereira LOR, Moreira RB, de Oliveira MP, Reis SO, de Oliveira Neto MP, Pirmez C. Is Leishmania (Viannia) braziliensis parasite load associated with disease pathogenesis? Int J Infect Dis. 2017;57:132-7.

11.	Weirather JL, Jeronimo SM, Gautam S, Sundar S, Kang M, Kurtz MA, et al. Serial quantitative PCR assay for detection, species discrimination, and quantification of Leishmania spp. in human samples. J Clin Microbiol. 2011;49(11):3892-904.

12.	Schallig H, Hu RVP, Kent AD, van Loenen M, Menting S, Picado A, et al. Evaluation of point of care tests for the diagnosis of cutaneous leishmaniasis in Suriname. BMC Infect Dis. 2019;19(1):25.

13.	Control of Leishmaniasis Geneva: World Health Organization 2010 March 22-26.

14.	Lopez M, Inga R, Cangalaya M, Echevarria J, Llanos-Cuentas A, Orrego C, et al. Diagnosis of Leishmania using the polymerase chain reaction: a simplified procedure for field work. American Journal of Tropical Medicine and Hygiene. 1993;3(49):348-56.

15.	Tsukayama P, Nunez JH, De Los Santos M, Soberon V, Lucas CM, Matlashewski G, et al. A FRET-based real-time PCR assay to identify the main causal agents of New World tegumentary leishmaniasis. PLoS Negl Trop Dis. 2013;7(1):e1956.

16.	Malhotra R, Indrayan A. A simple nomogram for sample size for estimating sensitivity and specificity of medical tests. Indian J Ophthalmol. 2010;58:519-22.

# **Supporting Information**

**S1 File 1. Supplemental Methods.**

**(DOCX)**

**S1 Table. Microscopy by PCR for CL *Detect*^TM^ Rapid Test (Collected by Dental Broach and Collected by Scraping) Positive and negative Subjects**

**(????)**

**S2 Table. Comparison of Results of All Studies by Different Assay Conditions**

**(DOCX)**

**xxxx**
